# Blinking Networks of Memristor Oscillatory Circuits in the Flux-Charge Domain

**DOI:** 10.3389/fnins.2021.618607

**Published:** 2021-04-22

**Authors:** Valentina Lanza, Jacopo Secco, Fernando Corinto

**Affiliations:** ^1^LMAH, FR-CNRS-3335, Université Le Havre Normandie, Le Havre, France; ^2^Department of Electronics, Politecnico di Torino, Turin, Italy

**Keywords:** neural network, biologically plausible neural networks, memristor, complex dynamics, nonlinear oscillators

## Abstract

Multistability phenomena and complex nonlinear dynamics in memristor oscillators pave the way to obtain efficient solutions to optimization problems by means of novel computational architectures based on the interconnection of single–device oscillators. It is well-known that topological properties of interconnections permit to control synchronization and spatio–temporal patterns in oscillatory networks. When the interconnections can change in time with a given probability to connect two oscillators, the whole network acts as a complex network with blinking couplings. The work of has shown that a particular class of *blinking complex networks* are able to completely synchronize in a faster fashion with respect to other coupling strategies. This work focuses on the specific class of blinking complex networks made of Memristor–based Oscillatory Circuits (MOCs). By exploiting the recent Flux–Charge Analysis Method, we make clear that synchronization phenomena in blinking networks of memristor oscillators having stochastic couplings, i.e., Blinking Memristor Oscillatory Networks (BMONs), correspond to global periodic oscillations on invariant manifolds and the effect of a blinking link is to shift the nonlinear dynamics through the infinite (invariant) manifolds. Numerical simulations performed on MOCs prove that synchronization phenomena can be controlled just by changing the coupling amongst them.

## 1. Introduction

Synchronization of complex networks with interacting units is an active research topic with applications in many fields such as engineering, computer sciences, neural networks (both biological and not), neuromorphic circuit computing and information security. Arenas et al. (2008) formulated a comprehensive review in 2008, describing the impacts of this field of research. One of the most challenging problem in complex network is the study of spatio–temporal patterns emerging from synchronization phenomena and their relationship with the network topology and interactions' dynamics among the (both oscillatory and/or chaotic) network units (Pecora and Carroll, 1998; Belykh V. N. et al., 2004; Boccaletti et al., 2006).

In literature great attention has been paid to investigate synchronization of complex networks with fixed topology and constant couplings. Only in recent years researchers have begun to focus on time–varying coupling strengths, due to the greater resemblance of this type of networks to real world systems. Different approaches have been adopted to study synchronization properties of these complex dynamical networks. For instance, Wu and Chen (2008) have provided several criteria of global synchronization, in the case of linearly coupled networks with continuous time-varying coupling. Following a complex networked control systems approach, De Lellis et al. (2008) and DeLellis et al. (2009) have considered time-varying adaptive couplings that evolve with respect to the difference among the oscillators. Several other authors (Berner et al., 2019a,b; Menara et al., 2019; Feketa et al., 2020) have focused on cluster synchronization in networks of phase oscillators with adapting coupling based on the dynamical change of the coupling strength between the oscillators. Moreover, Berner et al. (2019a,b) explored how such complex systems behave by “splitting the networks” into different clusters of nonlinear oscillators grouped in such a way that all the oscillators in a poll have the same frequency. For the scope of this work it is also necessary to take into account the dynamics of networks formed by memristors, in particular the work by Ascoli et al. (2015). In their work the authors extensively studied the synchronization dynamics between two memristor-coupled Hindmarsh-Rose oscillatory neural cells through theoretical investigation and numerical verification. On the other hand, by exploiting the mathematical tools from the control theory of switched systems, Stilwell et al. (2006) have studied a network of oscillators with switching topology, in order to model networks where some connections may fail or may create over time. In their work they have demonstrated that complex network can synchronize even when the topology is time-varying and instantaneously disconnected. Their thesis stands, though, on two pillars that serve as a *condicio sine qua non*: first the different circuits must be able to synchronize in a static time-average fashion; secondly the transition between two different topology must be sufficiently fast. Synchronization criteria in the case of complex network with arbitrary switching topology and strategies for designing switching signals to obtain synchronization have been investigated by Zhao et al. (2009).

A particular class of complex networks with switching topology permits to introduce the concept of blinking complex network (Belykh I. V. et al., 2004; Hasler and Belykh, 2005; Hasler et al., 2013a,b) in which the topology evolves in time according to a stochastic process. In particular, blinking networks have the peculiar feature that, starting by a fixed topology (called *pristine network*), new "long–range links" between two network units are randomly added according to a fixed probability. Once the new topological configuration is set, it will remain constant for a given amount of time, then again new links are randomly created, and so on. Belykh I. V. et al. (2004) have demonstrated that such time–varying random switching (i.e., blinking) topology induces complete synchronization in the networks. By creating randomly selected couplings, the chance of creating interconnected shortcuts increases, lowering the synchronization threshold sensibly.

The study and the development of blinking complex networks finds its importance especially in boosting functional properties of bio–inspired and neuromorphic circuits acting on the topology of synaptic connections. Neuromorphic circuits made of (1 or 2-d) arrays of silicon neurons exploits its analog nature, among other architectural characteristics, to enhance their computational efficiency and speed, but one of the main drawback is the necessity of a very high number of wired connections between the computational elements (e.g., large numbers of synapses is also used to overcome low resolution in synaptic weights—see also Chicca et al., 2014). Strategies that involve solely the reduction of the required connections, for instance reshaping the network topology adding a sum cell which has inputs and outputs that are not connected to all cells has been exploited by Seiler and Nossek (1993). This approach has permitted to reduce the number of connections for a Winner Take All architecture, however it did not take into account that most of the connections had to cross a wide part of the circuit, thus still rendering it not feasible. A more effective approach to this is to generate long distance connections, activated and deactivated randomly, in such a way that the network computations performance is the same as that of a corresponding locally–connected non–switching (averaged) network. The successful use of blinking/switching networks for solving this kind of issue has been proven, for example in a similar case to the one introduced by Seiler et al., by means of a Winner Take All locally–connected architecture (Devoe and Devoe, 2012). These results highlight the importance of the study of the synchronization dynamics of blinking networks since they can be one representative of the feasibility factors of real world neuromorphic computation.

This work focuses on Blinking Memristor Oscillatory Networks (BMONs) made of memristor–based nonlinear oscillators interacting via blinking couplings. A thorough study of nonlinear dynamics and bifurcation phenomena in memristor oscillators have been recently carried out by means of the Flux-Charge Analysis Method (FCAM). Such circuit methodology permits to show that the state space in the (voltage–current)–domain [i.e., (*v*−*i*)-domain] can be decomposed into invariant manifolds, where the dynamical behavior of the memristor oscillator may correspond to different complex attractors. In addition, bifurcation phenomena can be induced either by parameters on a fixed manifold (as in the classical theory of dynamical systems) or by changing the invariant manifold but with fixed parameters (a.k.a. *bifurcation without parameters*).

The ultimate goal of the manuscript is to show that blinking couplings in complex networks of memristor oscillators induce bifurcations without parameters so that the whole dynamics of a BMON takes place on an invariant manifold where periodic oscillations correspond to synchronized states. In this manuscript analysis of nonlinear dynamics and numerical simulations are focused on a specific BMON in which each oscillator is a third–order dynamical system describing the Chua's circuit with a memristor replacing the nonlinear resistor, but the FCAM provides the theoretical framework to grasp the role of network topology in BMONs including Memristor–based Oscillator Circuit (MOCs) of any order. It is also worth noting that bifurcation without parameters, and then the shift of nonlinear dynamics from one manifold to another, can be obtained by applying suitable charges or flux sources via time-varying voltage and/or current pulses with finite time duration (Corinto and Forti, 2017b). This work shows that BMONs exhibit analogous nonlinear phenomena without any external input. Moreover, we show that for memristor oscillators not having all the same initial conditions it is possible to change the invariant manifold on which the nonlinear dynamics takes place only by acting on the coupling topology. Our numerical investigations of BMONs shows how the creation of time–varying long–range random connections results into switching between invariant manifolds and then into synchronization.

At first, a brief introduction to the memristor oscillators is presented showing the internal dynamics and the numerical formulations of such systems in the flux-charge domain. Secondly it will be shown how the change of the topology of interacting memristor oscillators affects invariant manifolds on which nonlinear dynamics occur. Finally, synchronization phenomena in BMONs are analyzed via numerical simulations.

## 2. Problem Setup and Background

Let us briefly recall some results obtained in Corinto and Forti (2017a) for a MOC in the flux-charge domain. The nonlinear oscillator consists in a modified version of the well-known Chua's circuit where the nonlinear resistor is replaced by a memristor. The cited paper details and extends the results obtained in Corinto et al. (2011) For further details regarding the circuit model and its analysis please refer to Corinto and Forti (2017a). The State Equations (SEs) in dimensionless form^1^ are the following (*t*≥*t*_0_):

(1) $$\begin{array}{l} \frac{dx\left(t\right)}{dt}=\alpha \left(-x\left(t\right)+y\left(t\right)-n\left(x\left(t\right)\right)\right)+X_{0}\\ \frac{dy\left(t\right)}{dt}=x\left(t\right)-y\left(t\right)+z\left(t\right)\\ \frac{dz\left(t\right)}{dt}=-\beta y\left(t\right) \end{array}$$

with the initial conditions

$$\begin{array}{l} x\left(t_{0}\right)=\varphi_{M}\left(t_{0}\right)\\ y\left(t_{0}\right)=\varphi_{L}\left(t_{0}\right)\\ z\left(t_{0}\right)=\varphi_{L}\left(t_{0}\right)-\varphi_{M}\left(t_{0}\right)+Rq_{C_{2}}\left(t_{0}\right). \end{array}$$

where φ_*M*_(*t*_0_) is the initial flux across the memristor, φ_*L*_(*t*_0_) is the initial flux across the inductor, whereas *q*_*C*_2__(*t*_0_) is the initial charges of capacitor 2. Moreover, we have

(2) $$\begin{array}{r} n\left(x\right)=Rf\left(x\left(t\right)\right)=R\left(-\frac{8}{7}x+\frac{4}{63}x^{3}\right) \end{array}$$

and

(3) $$\begin{array}{r} X_{0}=\alpha \left(n\left(\varphi_{M}\left(t_{0}\right)\right)+\varphi_{M}\left(t_{0}\right)+RC_{1}v_{C_{1}}\left(t_{0}\right)-Li_{L}\left(t_{0}\right)\right). \end{array}$$

Readers interested in the circuit–theoretic definitions of the state variables, parameters and circuit analysis can refer to the study reported in Corinto and Forti (2017a).

Hereinafter and throughout the paper we set the following initial states and parameters:

(4) $$\begin{array}{r} \varphi_{L}\left(t_{0}\right)=Li_{L}\left(t_{0}\right)=0,\;v_{C_{1}}\left(t_{0}\right)=0,\;C_{1}=1,\;R=1. \end{array}$$

As shown in Corinto and Forti (2017a), the state space of the MOC in the (*v, i*)-domain can be decomposed in infinitely many three-dimensional manifolds $M\left(Q_{0}\right)$ defined, for any *Q*_0_ ∈ ℝ as

$$M\left(Q_{0}\right)=\left\{\text{w}={\left(v_{C_{1}},v_{C_{2}},i_{L},\varphi_{M}\right)}^{T}\in {\mathbb{R}}^{4}|Q\left(\text{w}\right)=Q_{0}\right\},$$

with

(5) $$\begin{array}{r} Q\left(\text{w}\right)=f\left(\varphi_{M}\right)+\frac{1}{R}\varphi_{M}+C_{1}v_{C_{1}}-\frac{L}{R}i_{L}. \end{array}$$

Furthermore, these manifolds are positive invariant for the dynamics of the MOC in the (*v, i*)–domain. Therefore, at instant *t* = *t*_0_ the system is on the manifold $M\left(Q_{0}\right)$, with *Q*_0_ = *Q*(**w**(*t*_0_)) and there it stays. The value of *Q*(**w**(*t*_0_)) depends on the memristor initial conditions and the circuit parameters.

** Proposition 1**. *Let us suppose the parameters and initial states as in (4) and φ_*M*_(*t*_0_)* ∈ {−1.5, 0, 1.5}. *Therefore, Q*(**w**(*t*_0_)) = 0, *that is the nonlinear dynamics of the MOC is on the so-called zero-manifold M(0). Thus, every trajectory defined by [*x*(*t*), *y*(*t*), *z*(*t*)] evolves on a three–dimensional space M(0) embedded in ℝ*^4^.

**P****roof**. According to the definition of *f* in (2), it is easy to see that $f\left(x\right)+\frac{x}{R}=f\left(x\right)+x=0$ for all *x* ∈ {−1.5, 0, 1.5}. Therefore, by using (4) and the definition of *Q* in (5), we deduce:

$$Q\left(\text{w}\left(t_{0}\right)\right)=f\left(\varphi_{M}\left(t_{0}\right)\right)+\frac{1}{R}\varphi_{M}\left(t_{0}\right)+C_{1}v_{C_{1}}\left(t_{0}\right)-\frac{L}{R}i_{L}\left(t_{0}\right)=0.$$

By extending (1), let us consider a one-dimensional array of *N* diffusively^2^ coupled MOCs described in the flux–charge domain by (*i* = 1, …, *N*):

(6) $$\begin{array}{l} \frac{dx_{i}\left(t\right)}{dt}=\alpha \left(-x_{i}\left(t\right)+y_{i}\left(t\right)-n\left(x_{i}\left(t\right)\right)\right)+X_{c_{0,i}}\\ \;+\;\underset{k\in N_{i}}{\sum }d_{ik}\left(x_{k}\left(t\right)-x_{i}\left(t\right)\right)\\ \frac{dy_{i}\left(t\right)}{dt}=x_{i}\left(t\right)-y_{i}\left(t\right)+z_{i}\left(t\right)\\ \frac{dz_{i}\left(t\right)}{dt}=-\beta y_{i}\left(t\right) \end{array}$$

where $N_{i}$ is the sphere of influence of *ith* MOC and $d_{ik}=\frac{\alpha }{R_{ik}}$ with *d*_*ik*_ = *d*_*ki*_. Following the same procedure for *X*_0_ in (3), the parameters (4) permit to derive

(7) $$\begin{array}{r} X_{c_{0,i}}=\alpha \left(n\left(\varphi_{M,i}\left(t_{0}\right)\right)+\varphi_{M,i}\left(t_{0}\right)\right)-\underset{k\in N_{i}}{\sum }d_{ik}\left(\varphi_{M,k}\left(t_{0}\right)-\varphi_{M,i}\left(t_{0}\right)\right). \end{array}$$

Analogously as in the single MOC, it is possible to prove that the state space of the Network of Memristor-based Oscillatory Circuits (NMOCs) in the (*v, i*)-domain is foliated by an infinite number of 3*N*–dimensional manifolds defined, for any ${\text{Q}}_{0}=\left(Q_{0,1},\ldots ,Q_{0,N}\right)\in {\mathbb{R}}^{N}$ as

$$M_{c}\left({\text{Q}}_{0}\right)=\left\{{\text{w}}_{c}\in {\mathbb{R}}^{4N}|Q_{i}\left({\text{w}}_{c}\right)=Q_{0,i},i=1,\ldots ,N\right\},$$

with **w**_*i*_ = (*v*_*C*_1_, *i*_, *v*_*C*_2_, *i*_, *i*_*L,i*_, φ_*M,i*_), ${\text{w}}_{c}={\left({\text{w}}_{1},\ldots ,{\text{w}}_{N}\right)}^{T}$ and

(8) $$\begin{array}{r} Q_{i}\left({\text{w}}_{c}\right)=f\left(\varphi_{M,i}\right)+\frac{1}{R}\varphi_{M,i}+C_{1}v_{C_{1},i}-\frac{L}{R}i_{L,i}-\underset{k\in N_{i}}{\sum }\frac{1}{R_{ik}}\left(\varphi_{M,k}-\varphi_{M,i}\right), \end{array}$$

From (7) and (8), we easily deduce that

(9) $$\begin{array}{r} X_{c_{0},i}=\alpha RQ_{i}\left({\text{w}}_{c}\right)\;\forall i=1,\ldots ,N \end{array}$$

In the next sections we exploit NMOCs described by (6) and (7) in the flux–charge domain to make clear the influence of the couplings *d*_*ik*_ on synchronization phenomena. In particular, by assuming that *d*_*ik*_ describes blinking links added to a pristine complex network (i.e., an underlying networks with fixed topology) then the NMOC acts as a BMON.

## 3. Coupling Effects on Invariant Manifolds Embedding Nonlinear Dynamics

Nonlinear dynamics and global attractors of a single MOC described by (1) with *X*_0_ in (3) can be controlled by applying suitable time-varying voltage and/or current pulses with finite time duration (Corinto and Forti, 2017b). The concept of bifurcation without parameters is the crucial tool to enable the programming of nonlinear dynamics via invariant manifolds. For instance, a sequence of period–doubling bifurcations (without parameters) leading from a periodic oscillation to a chaotic attractor can be induced by means of pulses applied to the MOC; each pulse has the effect of shifting the trajectory (due to the change of *X*_0_) from an invariant manifold on which the MOC exhibits a periodic behavior to an invariant manifold with a double–scroll chaotic attractor (Corinto and Forti, 2017b). In other words, bifurcations without parameters make possible to tune nonlinear dynamics of a MOC by selecting via pulses an invariant manifold embedding all the attractors.

This section shows how nonlinear dynamics and invariant manifolds of NMOCs described by (6) and (7) are influenced by the coupling terms *d*_*ik*_ (i.e., by the network topology).

First of all, it is possible to see that when all the memristors have the same initial conditions, the coupling terms *d*_*ik*_ play a role on the dynamics of the network [since they are present on the network Equations (6)], but they do not influence the manifold embedding the dynamics of the whole NMOC.

** Proposition 2**. *Let us consider the NMOC (6). If all the initial conditions of memristors are the same [i.e., φ_*M,i*_(*t*_0_) = φ_0_, ∀*i* = 1, …, *N*], then the attractors of the NMOC belong to an invariant manifold that is independent on the coupling parameters *d*_*ik*_*.

**P****roof**. It is easy to notice that when the initial conditions of each memristor are the same, that is φ_*M,i*_(*t*_0_) = φ_0_ for all *i* = 1, …, *N*, then

$$\underset{k\in N_{i}}{\sum }d_{ik}\left(\varphi_{M,k}\left(t_{0}\right)-\varphi_{M,i}\left(t_{0}\right)\right)=0.$$

Moreover, we have

(10) $$\begin{array}{l} Q_{i}\left({\text{w}}_{c}\left(t_{0}\right)\right)=f\left(\varphi_{M,i}\left(t_{0}\right)\right)+\frac{1}{R}\varphi_{M,i}\left(t_{0}\right)+C_{1}v_{C_{1},i}\left(t_{0}\right)-\frac{L}{R}i_{L,i}\left(t_{0}\right)\\ \;-\underset{k\in N_{i}}{\sum }\frac{1}{R_{ik}}\left(\varphi_{M,k}\left(t_{0}\right)-\varphi_{M,i}\left(t_{0}\right)\right)\\ \;=f\left(\varphi_{M,i}\left(t_{0}\right)\right)+\frac{1}{R}\varphi_{M,i}\left(t_{0}\right)+C_{1}v_{C_{1},i}\left(t_{0}\right)-\frac{L}{R}i_{L,i}\left(t_{0}\right)\\ \;=Q\left({\text{w}}_{i}\left(t_{0}\right)\right),\;i=1,\ldots ,N \end{array}$$

with *Q* defined as in (5). Therefore, the invariant manifold $M_{c}\left({\text{Q}}_{0}\right)$ is independent on the coupling.

Thus, in order to induce bifurcations without parameters by means of the couplings and then change the invariant manifold embedding the attractors of the NMOC, at least one initial condition of the memristors has to be different. Without loss of generality, let us suppose that the memristor initial condition in the first oscillator is different with respect to the others. Therefore, we can prove the following result:

** Proposition 3**. *Let us consider the NMOC (6) with the memristor initial conditions*:

$$\begin{array}{l} \varphi_{M,i}\left(t_{0}\right)=\varphi_{0}\;\forall i\neq 1\\ \varphi_{M,1}\left(t_{0}\right)={\tilde{\varphi }}_{0}\neq \varphi_{0}. \end{array}$$

*Let us suppose that the global dynamics of the NMOC evolves on the manifold* $M_{c}\left({\text{Q}}_{0}\right)$, *with* **Q**(**w**_*c*_(*t*_0_)) = **Q**_0_ = (*Q*_0,1_, …, *Q*_0,*N*_) *defined as in (8)*.

**Figure 1:**
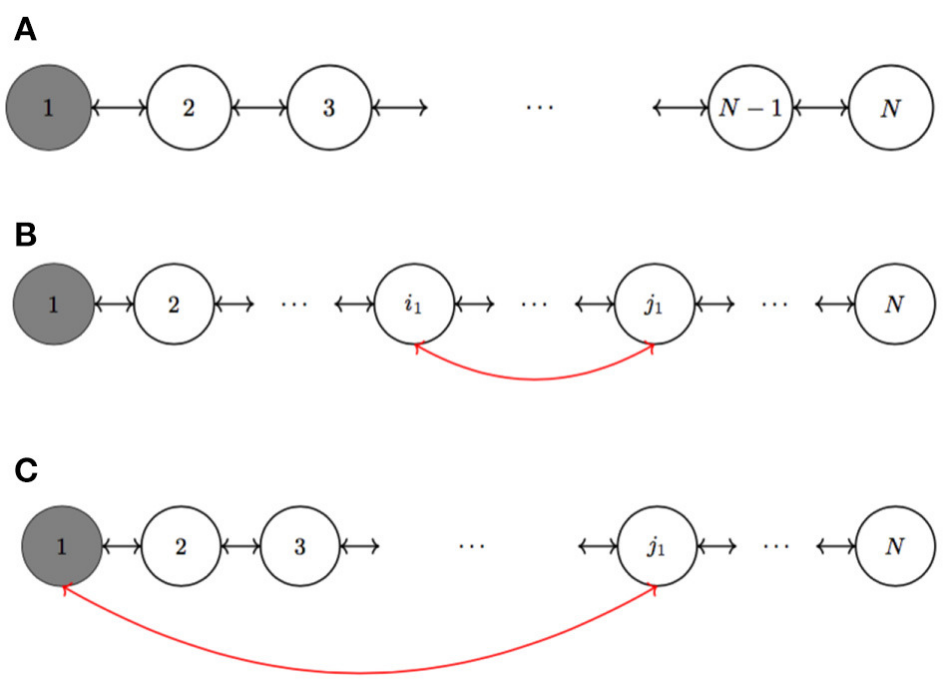
Network of *N* coupled MOCs. **(A)** For simplicity, we suppose a sphere of influence of radius one and zero boundary conditions. The memristor initial state of the first MOC is different with respect of the other ones. When we add a link between node *i*_1_ and node *j*_1_ ≠ 1, we have two possibilities: either **(B)** *i*_1_ ≠ 1 or **(C)** *i*_1_ = 1.

*Moreover, let us suppose to add a link between the nodes *i*_1_ and *j*_1_, that are not yet linked. Therefore, we have the following two cases*:

- *if *i*_1_ ≠ 1 and *j*_1_ ≠ 1, as in Figure 1B, the NMOCs Equations (6) are modified but the dynamics of the network still evolves on the manifold* $M_{c}\left({\text{Q}}_{0}\right)$
- *if *i*_1_ = 1, as in Figure 1C, not only the NMOCs Equations (6) change as in the previous case, but the nonlinear dynamics of the NMOC shift from the invariant manifold* $M_{c}\left({\text{Q}}_{0}\right)$ *to the manifold* $M_{c}\left({\text{Q}}_{0}^{\prime }\right)$, *where* ${\text{Q}}_{0}^{\prime }=\left(Q_{0,1}^{\prime },\ldots ,Q_{0,N}^{\prime }\right)$ *with*
  $$\begin{array}{l} Q_{0,1}^{\prime }=Q_{0,1}-\frac{1}{R_{1,j_{1}}}\left(\varphi_{M,j_{1}}\left(t_{0}\right)-\varphi_{M,1}\left(t_{0}\right)\right)=Q_{0,1}-\frac{1}{R_{1,j_{1}}}\Delta \varphi_{0}\\ Q_{0,j_{1}}^{\prime }=Q_{0,j_{1}}+\frac{1}{R_{1,j_{1}}}\Delta \varphi_{0}\\ Q_{0,i}^{\prime }=Q_{0,i}\;\forall i\neq \left\{1,j_{1}\right\}, \end{array}$$
  *and* $\Delta \varphi_{0}=\varphi_{0}-{\tilde{\varphi }}_{0}.$

**P****roof**. Let us suppose:

$$\begin{array}{l} \varphi_{M,i}\left(t_{0}\right)=\varphi_{0}\;\forall i\neq 1\\ \varphi_{M,1}\left(t_{0}\right)={\tilde{\varphi }}_{0}\neq \varphi_{0} \end{array}$$

and $\Delta \varphi_{0}=\varphi_{0}-{\tilde{\varphi }}_{0}.$

In this case, the NMOCs Equation (6) become:

(11) $$\begin{array}{l} \frac{dx_{i}\left(t\right)}{dt}=\alpha \left(-x_{i}\left(t\right)+y_{i}\left(t\right)-n\left(x_{i}\left(t\right)\right)\right)+X_{c_{0,i}}\\ \;+\;\underset{k\in N_{i}}{\sum }d_{ik}\left(x_{k}\left(t\right)-x_{i}\left(t\right)\right)\\ \frac{dy_{i}\left(t\right)}{dt}=x_{i}\left(t\right)-y_{i}\left(t\right)+z_{i}\left(t\right)\\ \frac{dz_{i}\left(t\right)}{dt}=-\beta y_{i}\left(t\right) \end{array}$$

with

(12) $$X_{c_{0,i}}=\;\left\{ \begin{array}{l} \alpha (n({\tilde{\varphi }}_{0})+{\tilde{\varphi }}_{0})-\sum_{k\in N_{i}}d_{ik}\Delta \varphi_{0},\text{ if }i=1\;\\ \alpha (n({\tilde{\varphi }}_{0})+{\tilde{\varphi }}_{0})+d_{1i}\Delta \varphi_{0}\text{ if }i\in N_{1}:i\neq 1\\ \alpha (n(\varphi_{0})+\varphi_{0}),\text{ if }i\notin N_{1} \end{array}\right.\;$$

Moreover, according to (8), for any *t*≥*t*_0_ the global dynamics of the NMOC (11) evolves on the manifold $M_{c}\left({\text{Q}}_{0}\right)$, with **Q**(**w**_*c*_(*t*_0_)) = **Q**_0_ = (*Q*_0,1_, …, *Q*_0,*N*_) and

$$\begin{array}{l} Q_{0,1}=f(\varphi_{M,1})+\frac{1}{R}\varphi_{M,1}(t_{0})\\ \;-\;\sum_{k\in N_{1}}\frac{1}{R_{1k}}(\varphi_{M,k}(t_{0})\\ \;-\varphi_{M,1}(t_{0}))\\ \;=f({\tilde{\varphi }}_{0})+\frac{1}{R}{\tilde{\varphi }}_{0}-\sum_{k\in N_{1}}\frac{1}{R_{1k}}\Delta \varphi_{0}\\ \forall i\in N_{1}:i\neq 1,\;Q_{0,i}=f(\varphi_{M,i})+\frac{1}{R}\varphi_{M,i}(t_{0})\\ \;-\;\sum_{k\in N_{i}}\frac{1}{R_{ik}}(\varphi_{M,k}(t_{0})\\ \;-\varphi_{M,i}(t_{0}))\\ \;=f(\varphi_{0})+\frac{1}{R}\varphi_{0}+\frac{1}{R_{1i}}\Delta \varphi_{0}, \end{array}$$

(13) $$\begin{array}{l} \forall i\notin N_{1}\;Q_{0,i}=f(\varphi_{M,i})+\frac{1}{R}\varphi_{M,i}(t_{0})-\sum_{k\in N_{i}}\frac{1}{R_{ik}}(\varphi_{M,k}(t_{0})\\ \;-\varphi_{M,i}(t_{0}))\\ \;=f(\varphi_{0})+\frac{1}{R}\varphi_{0}. \end{array}$$

Let us suppose now to add a link between the nodes *i*_1_ and *j*_1_, that are not yet linked. We can have two different cases (see Figure 1):

1. if *i*_1_≠1 and *j*_1_ ≠ 1, as in Figure 1B, the NMOCs Equations (6) are modified. Indeed, for the *x*-components of the MOCs with indexes *i*_1_ and *j*_1_ we have additional coupling terms due to the new link:
  $$\begin{array}{l} \frac{dx_{i_{1}}\left(t\right)}{dt}=\alpha \left(-x_{i_{1}}\left(t\right)+y_{i_{1}}\left(t\right)-n\left(x_{i_{1}}\left(t\right)\right)\right)+X_{c_{0,i_{1}}}\\ \;+\underset{k\in N_{i_{1}}}{\sum }d_{i_{1}k}\left(x_{k}\left(t\right)-x_{i_{1}}\left(t\right)\right)+d_{i_{1}j_{1}}\left(x_{j_{1}}\left(t\right)-x_{i_{1}}\left(t\right)\right) \end{array}$$
  (14) $$\begin{array}{l} \frac{dx_{j_{1}}\left(t\right)}{dt}=\alpha \left(-x_{j_{1}}\left(t\right)+y_{j_{1}}\left(t\right)-n\left(x_{j_{1}}\left(t\right)\right)\right)+X_{c_{0,j_{1}}}\\ \;+\underset{k\in N_{j_{1}}}{\sum }d_{j_{1}k}\left(x_{k}\left(t\right)-x_{j_{1}}\left(t\right)\right)+d_{i_{1}j_{1}}\left(x_{i_{1}}\left(t\right)-x_{j_{1}}\left(t\right)\right) \end{array}$$
  while the other equations do not change.
  It is important to notice that, since φ_*M*,_*i*__1__(*t*_0_) = φ_*M*,_*j*__1__(*t*_0_), the addition of the new link does not change the expression of *X*_*c*_0,_1__1___ and *X*_*c*_0,_*j*__1___. Therefore, for all *i* = 1, …, *N*, the *X*_*c*_0,*i*__ terms are still the same, and from the relation between *X*_*c*_0,*i*__ and *Q*_0,*i*_ in (9), we can deduce that the network still evolves on the manifold $M_{c}\left({\text{Q}}_{0}\right)$,
2. if *i*_1_ = 1, as in Figure 1C, not only the NMOCs Equations (6) change as in the previous case, but also the coefficients *X*_*c*_0,1__ and *X*_*c*_0,_*j*__1___. In fact, we have the new coefficients:
  $$\begin{array}{l} X_{c_{0,1}}^{\prime }=X_{c_{0,1}}-d_{1j_{1}}\Delta \varphi_{0}\\ X_{c_{0,j_{1}}}^{\prime }=d_{1j_{1}}\Delta \varphi_{0} \end{array}$$
  This implies that the nonlinear dynamics of the NMOC shift from the invariant manifold $M_{c}\left({\text{Q}}_{0}\right)$ to the manifold $M_{c}\left({\text{Q}}_{0}^{\prime }\right)$, where ${\text{Q}}_{0}^{\prime }=\left(Q_{0,1}^{\prime },\ldots ,Q_{0,N}^{\prime }\right)$ with
  $$\begin{array}{l} Q_{0,1}^{\prime }=Q_{0,1}-\frac{1}{R_{1,j_{1}}}\left(\varphi_{M,j_{1}}\left(t_{0}\right)-\varphi_{M,1}\left(t_{0}\right)\right)=Q_{0,1}-\frac{1}{R_{1,j_{1}}}\Delta \varphi_{0}\\ Q_{0,j_{1}}^{\prime }=Q_{0,j_{1}}+\frac{1}{R_{1,j_{1}}}\Delta \varphi_{0}, \end{array}$$
  while $Q_{0,i}^{\prime }=Q_{0,i}$ does not change for all *i* different from 1 and *j*_1_.

The next section shows the application of this analysis to the numerical study of synchronization phenomena in NMOCs and BMONs.

## 4. Spatio–Temporal Synchronization Phenomena

This section presents the numerical analysis of spatio–temporal synchronization phenomena in NMOCs in two specific cases of interest: (a) the addition of just one single link in order to make clear the effect of a connection on the manifold shift; (b) the inclusion of blinking interconnections having a given probability to connect two MOCs. As preliminary step, we show the effect of the memristor initial conditions in such NMOC with fixed diffusive topology.

### 4.1. NMOC With Fixed Diffusive Topology

Let us consider *N* = 4 MOCs forming a NMOC whose SEs are given by (6) and such that each MOC has an influence sphere of radius *r* = 1, that is $N_{i}=\left\{i-1,i,i+1\right\}$ for *i* = 1, …, 4. Moreover, zero boundary conditions are considered. We suppose that each oscillator has the following circuit parameters: α = 9.5 and β = 15. Moreover, let us consider as the *ith* MOC's initial conditions:

$$x_{i}\left(0\right)=\varphi_{0,i}\;y_{i}\left(0\right)=0\;z_{i}\left(0\right)=-\varphi_{0,i}+{\bar{\varphi }}_{0,i},$$

with

(15) $$\begin{array}{rc} \varphi_{0,1}={\tilde{\varphi }}_{0}=0 & \;{\bar{\varphi }}_{0,1}=Rq_{C_{2},1}\left(0\right)=1.1\\ \varphi_{0,i}=\varphi_{0}=-1.5 & \;{\bar{\varphi }}_{0,i}=Rq_{C_{2},i}\left(0\right)=-1\;\forall i\neq 1. \end{array}$$

Therefore, here Δφ_0_ = −1.5.

With this choice of memristor initial conditions, we have $n\left({\tilde{\varphi }}_{0}\right)+{\tilde{\varphi }}_{0}=n\left(\varphi_{0}\right)+\varphi_{0}=0$. Each uncoupled MOC (i.e., *d*_*ik*_ = 0) exhibits a double-scroll attractor belonging to the zero–manifold, since, as we have shown in Proposition 1, *Q*_0_ = 0 when φ_*M*_(*t*_0_) ∈ {−1.5, 0, 1.5}.

Moreover, with this choice of initial conditions, it follows that

(16) $$X_{c_{0,i}}=\left\{ \begin{array}{l} -\sum_{k\in N_{i}}d_{ik}\Delta \varphi_{0},\text{ if }i=1\\ +d_{1i}\Delta \varphi_{0}\text{ if }i\in N_{1}:i\neq 1\\ 0,\text{ if }i\notin N_{1}. \end{array}\right.$$

Hence, the nonlinear dynamics of the NMOC is not on the 3*N*-dimensional zero-manifold $M_{c}\left(0\right)$, but from (13) we deduce that the manifold of the NMOC is $M_{c}\left({\text{Q}}_{0}\right)$, with:

(17) $$X_{c_{0,i}}=\left\{ \begin{array}{l} -\sum_{k\in N_{i}}\frac{1}{R_{1k}}\Delta \varphi_{0},\text{ if }i=1\\ \frac{1}{R_{1i}}\Delta \varphi_{0}\text{ if }i\in N_{1}:i\neq 1\\ 0,\text{ if }i\notin N_{1}. \end{array}\right.$$

In the following, let us consider a fixed topology defined by a diffusive (space–invariant) couplings with *d*_*ik*_ = *d* = 0.05α. In this case, **Q**_0_ is the following:

(18) $$Q_{0,i}=\left\{ \begin{array}{l} -\sum_{k\in N_{1}}\frac{1}{R_{1k}}\Delta \varphi_{0}=-\frac{1}{R_{12}}\Delta \varphi_{0}=0.075\text{ if }i=1\\ \frac{1}{R_{12}}\Delta \varphi_{0}=-0.075,\text{ if }i=2\;\\ 0\text{ if }i\notin \{1,2\}. \end{array}\right.$$

**Figure 2:**
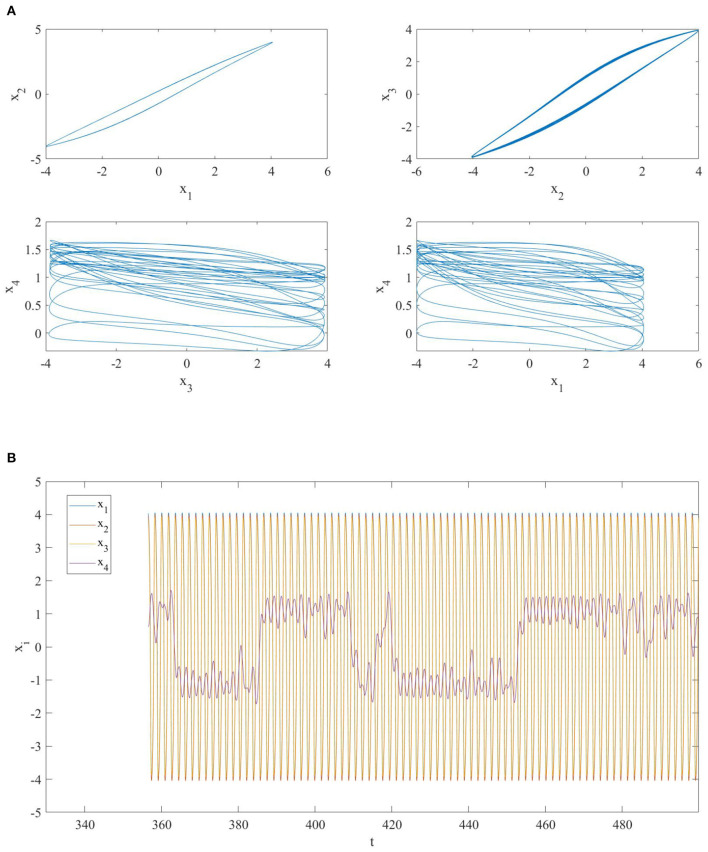
**(A)** Phase portrait and **(B)** time series of a network of four diffusively coupled MOCs. The oscillators are nearly in-phase synchronized except for the fourth one.

Thus, due to the choice of different memristor initial conditions and the coupling (diffusive topology), the global evolution of the NMOC is not on the zero-manifold and the first three oscillators synchronize nearly in–phase, while the last one is still chaotic (see Figure 2).

### 4.2. Addition of One Link

We are interested in investigating what happens when we add a link between the *i*_1_-th and the *j*_1_ MOCs of the NMOC having the fixed diffusive topology analyzed in the previous section. For this purpose, we consider the NMOC with fixed diffusive topology from *t* = 0 to *t* = 500, then we add a link at *t* = 500 with *d*_*i*_1_, *j*_1__ = *d* and analyze the dynamic behavior of the whole NMOC till *t* = 1, 000. Indeed, according to section 3, the SEs of the NMOC including the additional link change as in (14).

**Figure 3:**
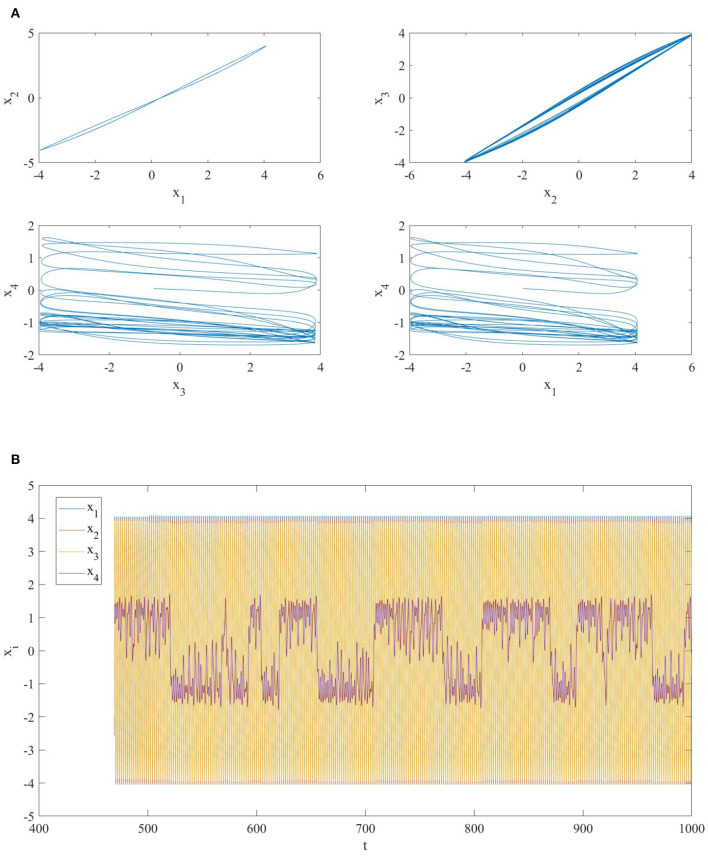
**(A)** Phase portrait and **(B)** time series of a network of four diffusively coupled MOCs when a link between the first and the third oscillator is added at *t* = 500. The oscillators in the network are not synchronized.

**Figure 4:**
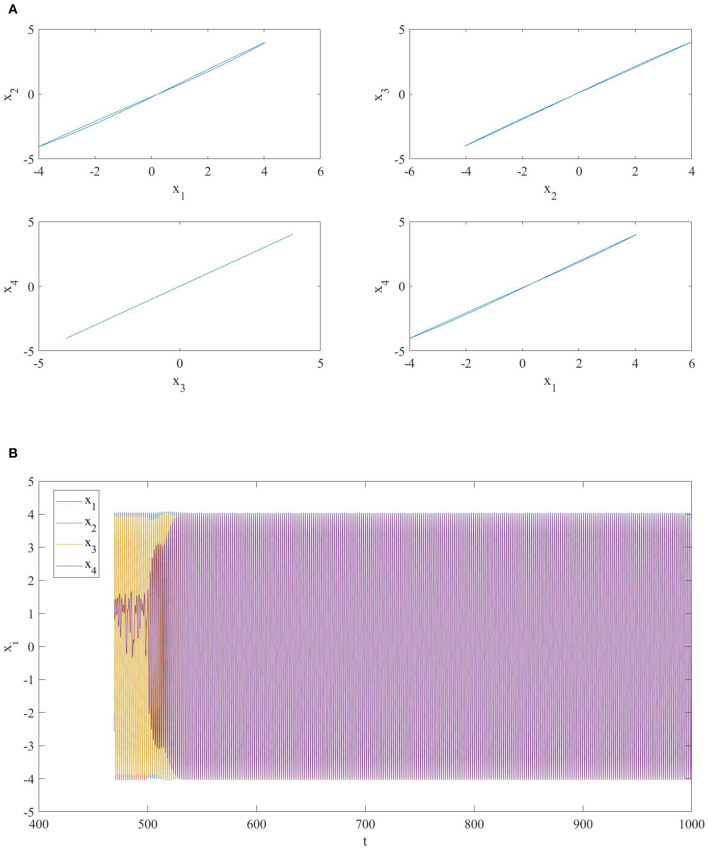
**(A)** Phase portrait and **(B)** time series of a network of four diffusively coupled MOCs when a link between the first and the fourth oscillator is added at *t* = 500. The addition of this link makes all the oscillators to synchronize in phase.

**Figure 5:**
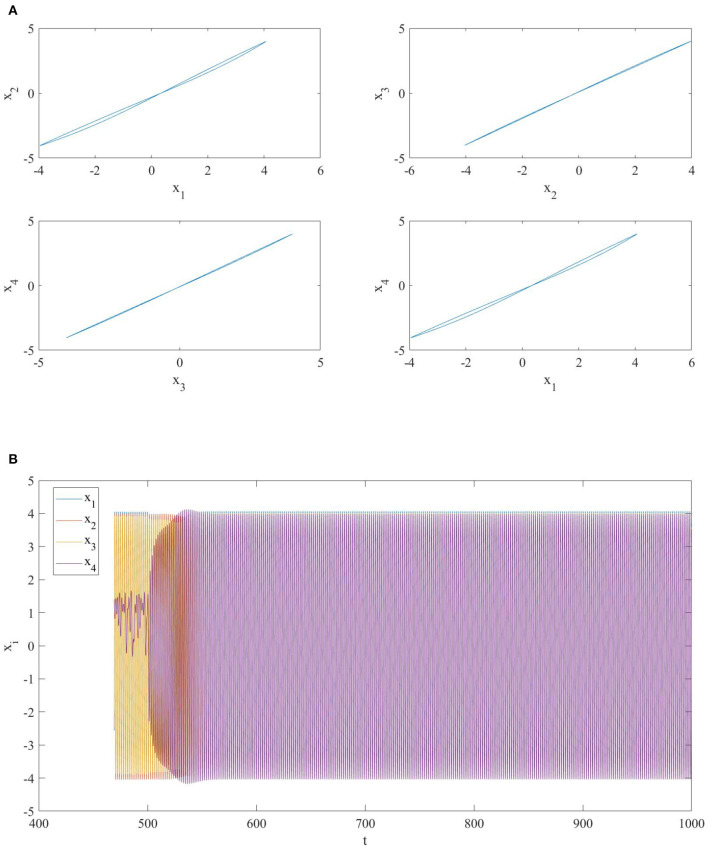
**(A)** Phase portrait and **(B)** time series of a network of four diffusively coupled MOCs when a link between the second and the fourth oscillator is added at *t* = 500. The addition of the new link makes the fourth MOC to synchronize with the others.

In the choice of *i*_1_ and *j*_1_, we have here three possibilities:

(a) *i*_1_ = 1 *and* *j*_1_ = 3: since these two MOCs have different memristor initial conditions, at *t* = 500 the nonlinear dynamics of the whole NMOC shifts from the invariant manifold $M_{c}\left({\text{Q}}_{0}\right)$ to $M_{c}\left({\text{Q}}_{0}^{\prime }\right)$, where ${\text{Q}}_{0}^{\prime }=\left(Q_{0,1}^{\prime },\ldots ,Q_{0,N}^{\prime }\right)$ with
  $$\begin{array}{l} Q_{0,1}^{\prime }=Q_{0,1}-\frac{1}{R_{1,3}}\Delta \varphi_{0}=0.15,\\ Q_{0,2}^{\prime }=Q_{0,2}=-0.075\\ Q_{0,3}^{\prime }=Q_{0,3}+\frac{1}{R_{1,3}}\Delta \varphi_{0}=-0.075, \end{array}$$
  $$\begin{array}{l} Q_{0,4}^{\prime }=Q_{0,4}=0. \end{array}$$
  In this case, the addition of the link does not modify the synchronization properties of the network (see Figure 3).
(b) *i*_1_ = 1 *and* *j*_1_ = 4: also in this case the memristor initial conditions are different, therefore the dynamic evolution of the NMOC for any *t*≥500 takes place on the invariant manifold $M_{c}\left({\text{Q}}_{0}^{\prime }\right)$, where ${\text{Q}}_{0}^{\prime }=\left(Q_{0,1}^{\prime },\ldots ,Q_{0,4}^{\prime }\right)$ with
  $$\begin{array}{l} Q_{0,1}^{\prime }=Q_{0,1}-\frac{1}{R_{1,4}}\Delta \varphi_{0}=0.15,\\ Q_{0,2}^{\prime }=Q_{0,2}=-0.075 \end{array}$$
  $$\begin{array}{l} Q_{0,3}^{\prime }=Q_{0,3}=0\\ Q_{0,4}^{\prime }=Q_{0,4}+\frac{1}{R_{1,4}}\Delta \varphi_{0}=-0.075. \end{array}$$
  As represented in Figure 4, the new link makes the fourth MOC to synchronize with the others, and then the whole NMOC exhibits in–phase synchronization among the memristor–based oscillator circuits.
(c) *i*_1_ = 2 *and* *j*_1_ = 4: in this case the two MOCs have identical memristor initial conditions, therefore the network remains on the manifold $M_{c}\left({\text{Q}}_{0}\right)$. As it is possible to see in Figure 5, the addition of the link gives rise to a (global) periodic state corresponding to a in–phase synchronization among the oscillators in the NMOC.

The three cases (a)–(c) makes clear that adding suitable links in NMOCs with fixed topology can induce synchronization phenomena. In particular, for the case under study the adding of a link connecting the fourth MOC is essential to obtain in-phase synchronization because it was out-of-phase in the MNMOC with fixed diffusive couplings. A more general case is considered in the next section by investigating larger NMOCs with fixed diffusive topology and where new links are added with a given probability and for a fixed time. We refer to such case as BMONs.

### 4.3. Blinking Memristor Oscillatory Networks (BMONs)

Several works in literature (Berner et al., 2019a,b; Menara et al., 2019; Feketa et al., 2020) have investigated networks of phase oscillators with adapting coupling based on the dynamical change of the coupling strength between the oscillators in order to obtain cluster synchronization. In the following we adopt the approach of Belykh I. V. et al. (2004), Hasler and Belykh (2005), and Hasler et al. (2013a,b) and we consider Blinking Memristor Oscillatory Networks, that is networks with a fixed topology (the so-called pristine network) and where new long-range links are randomly added and removed at each time period τ.

The Blinking Memristor Oscillatory Networks is defined as follows (*i* = 1, …, *N*):

(19) $$\begin{array}{l} \frac{dx_{i}\left(t\right)}{dt}=\alpha \left(-x_{i}\left(t\right)+y_{i}\left(t\right)-n\left(x_{i}\left(t\right)\right)\right)+X_{c_{0,i}}\\ \;+\overset{N}{\underset{k=1}{\sum }}d_{ik}\left(t\right)\left(x_{k}\left(t\right)-x_{i}\left(t\right)\right)\\ \frac{dy_{i}\left(t\right)}{dt}=x_{i}\left(t\right)-y_{i}\left(t\right)+z_{i}\left(t\right)\\ \frac{dz_{i}\left(t\right)}{dt}=-\beta y_{i}\left(t\right) \end{array}$$

where

$$X_{c_{0,i}}=\alpha (n(\varphi_{M,i}(t_{0}))+\varphi_{M,i}(t_{0})-\sum_{k=1}^{n}d_{ik}(t)(\varphi_{M,k}(t_{0})-\varphi_{M,i}(t_{0})).$$

Following Belykh I. V. et al. (2004), Hasler and Belykh (2005), Hasler et al. (2013a,b), once divided the time axis into intervals of length τ, we consider the coupling coefficients as follows:

$$d_{ik}(t)=\left\{ \begin{array}{l} d\text{ if }k\in N_{i}\\ ds_{ik}^{m}\text{ if }k\text{ is not included in }N_{i},\text{ and }(m-\text{1})\tau \\ \;<t<m\tau , \end{array}\right.$$

with $N_{i}=\left\{i-1,i,i+1\right\}$ (*i* = 1, …, *N*), *d* = 0.075α, for all *i*, and $s_{ik}^{m}$ equal to 1 with probability *p* and equal to 0 with probability 1−*p*. Also in this case, zero-boundary conditions are considered. Therefore, it means that the corresponding pristine network (whose links are always present for each *t*) is a diffusive MOC network where each oscillator has a sphere of influence of radius 1. In addition, new long-range links are added with a probability *p* and during time intervals of length τ. In the following, we consider a switching probability of *p* = 0.03 and τ = 0.1. It is possible to see, as shown also in Belykh I. V. et al. (2004), that adding long-range links improve the synchronizations properties of the network.

Here we have considered *N* = 30. Moreover, we suppose that each oscillator has the following circuit parameters: α = 9.5 and β = 15. As in the previous case, we suppose that all the oscillators have identical initial conditions except the first one:

$$x_{i}\left(0\right)=\varphi_{0,i}\;y_{i}\left(0\right)=0\;z_{i}\left(0\right)=-\varphi_{0,i}+{\bar{\varphi }}_{0,i},$$

with

(20) $$\begin{array}{rc} \varphi_{0,1}={\tilde{\varphi }}_{0}=0 & \;{\bar{\varphi }}_{0,1}=1.1\\ \varphi_{0,i}=\varphi_{0}=-1.5 & \;{\bar{\varphi }}_{0,i}=-1\;\forall i\neq 1. \end{array}$$

Thus, Δφ_0_ = −1.5.

**Figure 6:**
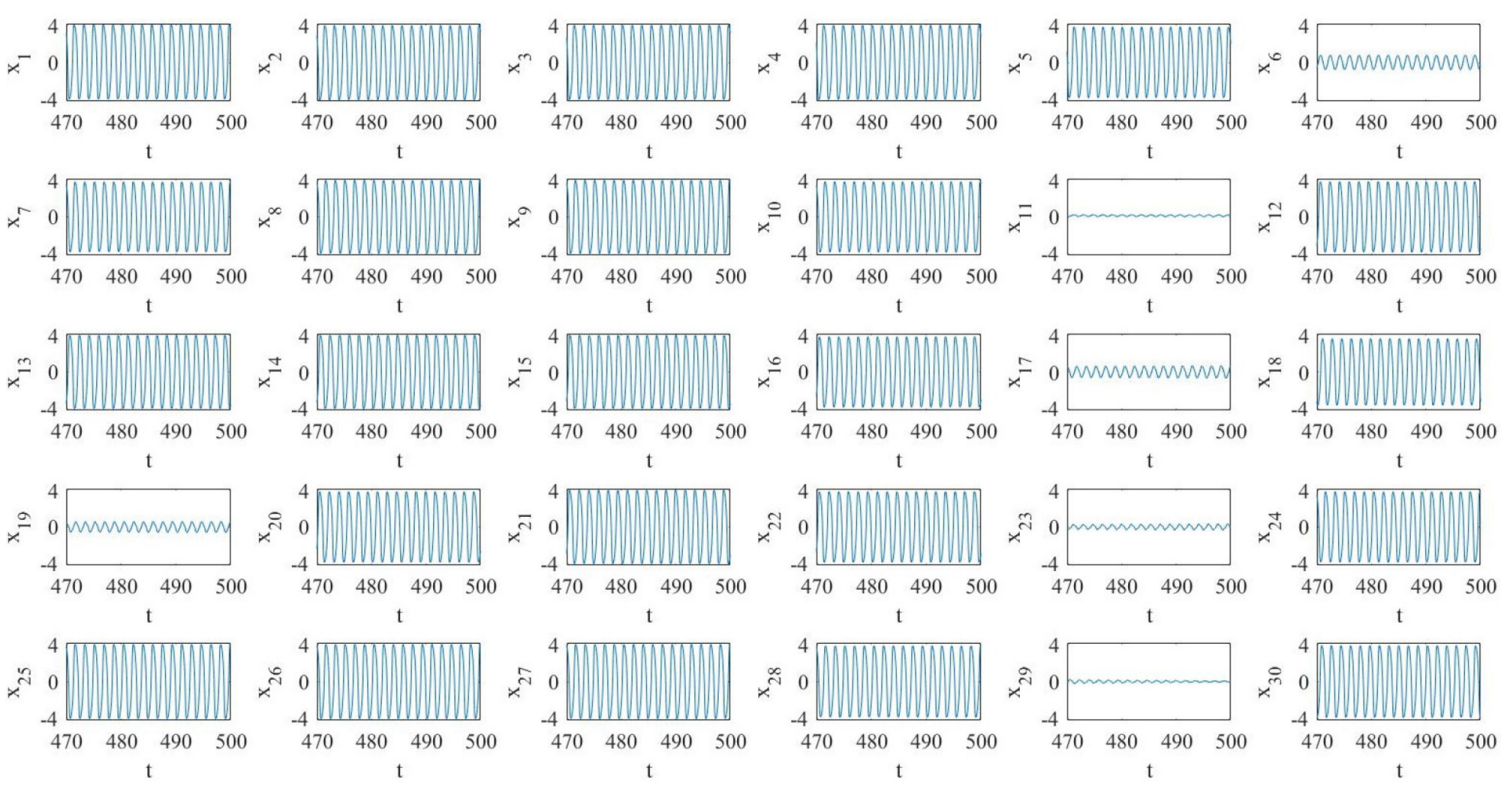
Pristine diffusive network of *N* = 30 MOCs. Several oscillators (but not all) are on the large amplitude limit cycle.

**Figure 7:**
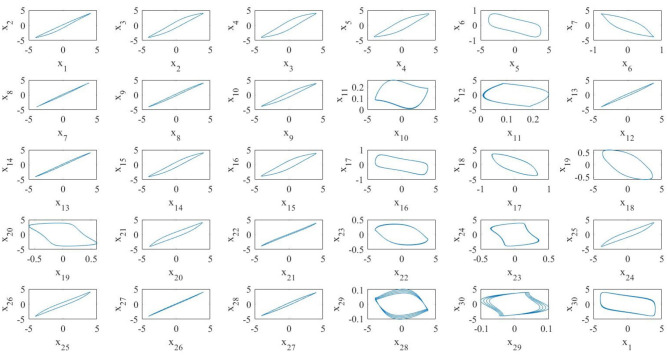
Phase portraits in the case of a diffusive array of *N* = 30 MOCs. Only some oscillators are synchronized.

The numerical simulations of the pristine (i.e., fixed topology) diffusive network, reported in Figure 6, show that the network dynamics is such that the majority of the oscillators are on the large limit cycle surrounding the double-scroll except few of them. Moreover, the oscillators are not synchronized, as it is possible to see in the phase portraits of Figure 7. It is interesting to notice that in this case the last MON is on the large limit cycle while in the previous diffusive network of four MOCs, the last one was chaotic. Since the boundary conditions for both the cases are the same, we presume that the different behavior is due to the number of oscillators in the two networks.

**Figure 8:**
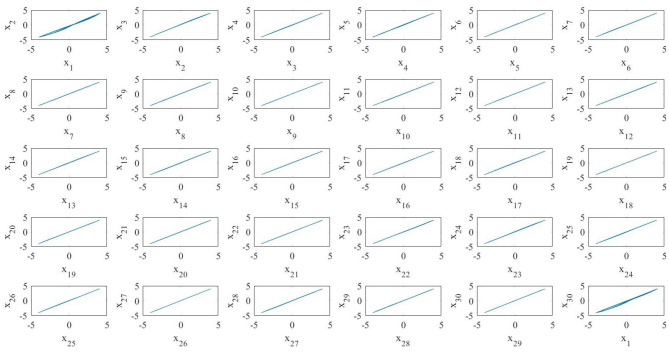
A realization of a blinking array of *N* = 30 MOCs. All the oscillators synchronize in phase.

Figure 8 shows a realization of the BMON (19). Thanks to the addition of time-dependent on–off long-range links the blinking network, unlike the pristine one, exhibits in-phase synchronization.

Let us investigate how the blinking topology affects the invariant manifold on which the BMON evolves. According to (17), the pristine network at time *t* = 0 is on the manifold $M_{c}\left({\text{Q}}_{0}\right)$ with

(21) $$Q_{0,i}=\left\{ \begin{array}{l} -\sum_{k\in N_{1}}\frac{1}{R_{1k}}\Delta \varphi_{0}=-\frac{1}{R_{12}}\Delta \varphi_{0}=0.1125\text{ if }i=1\\ \frac{1}{R_{12}}\Delta \varphi_{0}=-0.1125,\text{ if }i=2\\ 0\text{ if }i\notin \{1,2\}. \end{array}\right.$$

**Figure 9:**
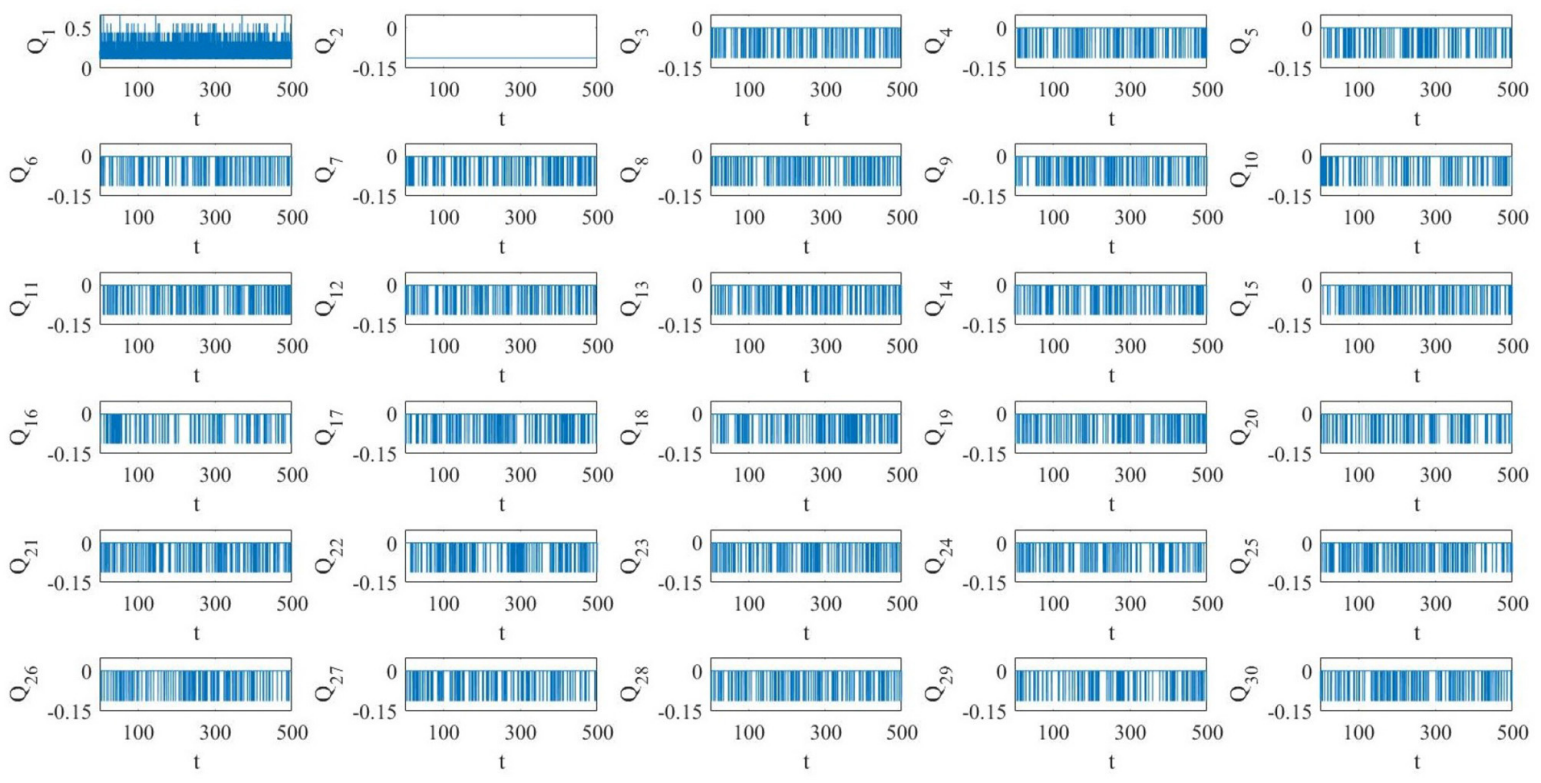
Components *Q*_*i*_ (*i* = 1, …, 30) of **Q**[**w**_*c*_(*t*)] as function of time for the blinking network under study. At each blink, new non-local links are added and the whole network possibly changes of manifold.

As seen in section 3, if a new link is added between two oscillators with different memristor initial conditions, the whole BMON changes of invariant manifold on which nonlinear attractors occur. Therefore, in our BMON, at each time we add a new long-range link, depending if it involves the first MOC or not, the whole BMON can switch from one manifold to another. In Figure 9, the components *Q*_*i*_(*t*) = *Q*_*i*_(**w**_*c*_(*t*)) (*i* = 1, …, 30) of **Q**(**w**_*c*_(*t*)) as function of time are represented. It is worth noting that these components are piece-wise constant since each manifold is positive invariant, so the system stays on one manifold till a new link (between MOCs with different memristor initial conditions) is added or removed. Clearly, the component *Q*_1_ is the one who switches the most, since it changes each time we add a link with the first MOC as edge destination. On the contrary, for *i*≥3, the components *Q*_*i*_ change only if the new links involve the first MOC and the *ith* MOC. As we have pointed out in section 3, if the two edges of the new link are different from 1, the BMON evolves on the same invariant manifold. Finally, the component *Q*_2_ does not change at all, since the link from the first and the second MOC (the only one who could make change this component) is already present in the pristine network. All the possible new links that involve the second MOC are among it and a *j*-th oscillator (with *j*≥4), but, since these MOCs have the same memristor initial conditions, the manifold embedding the evolution of the BMON does not change.

For further information we have also considered the average system whose SEs are the same as (19), with (for *i*≠*k*)

(22) $$d_{ik}\left(t\right)\text{=}\left\{ \begin{array}{l} d\text{ if nodes }i\text{ and }k\text{ are connected in the pristine}\\ \text{ network }\\ pd\text{ if not}. \end{array}\right.$$

**Figure 10:**
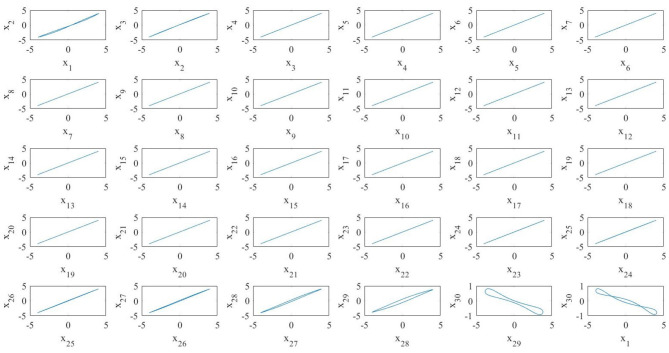
Phase portrait of the average system of *N* = 30 MOCs. All the oscillators, except the last one, synchronize in phase.

It is interesting to notice that in this case the average system does not exhibit in-phase synchronization because of the last MOC (see Figure 10). Indeed, the fact that the average BMON synchronizes is a sufficient but not necessary condition in order to have the synchronization of the blinking network (Belykh I. V. et al., 2004). Moreover, in our case, due to the different memristor initial conditions, the MOCs are not identical as in Belykh I. V. et al. (2004). Thus, as stated in the beginning of this work, we show that for memristor oscillators not having all the same initial conditions it is possible to change the invariant manifold on which the nonlinear dynamics takes place only by acting on the coupling topology. The numerical procedures that exploit the FCAM analysis method, of BMONs shows how the creation of time–varying long–range random connections results into switching between invariant manifolds which leads them to synchronization. Our results show that nonlinear dynamical effects generated by the blinking action of couplings and the consequent switching among invariant manifolds is needed in order to achieve a in-phase synchronization for the whole BMON.

## 5. Conclusion

In this paper we have considered a network of memristor nonlinear oscillators in the Flux-Charge Analysis Method (FCAM) framework, in order to show that it is possible to tune nonlinear dynamics of complex networks of memristor–based oscillators by simply adding new links among the oscillators, thus by acting on the blinking coupling topology. This has been done under the hypothesis of different memristor initial conditions. The FCAM permits to interpret the dynamical evolution of the whole blinking network of memristor oscillators as the periodic/chaotic attractors occurring on different invariant manifolds. Numerical simulation show that the synchronization properties can be improved by adding blinking long-range links and consequently by randomly switching of the invariant manifold.

## Data Availability Statement

The original contributions presented in the study are included in the article/supplementary material, further inquiries can be directed to the corresponding author/s.

## Author Contributions

VL and FC supervised and developed the mathematical model described in the paper. JS performed the simulations. FC wrote abstract and section 1. VL and JS wrote sections 3–7. All authors contributed in the analysis of the results and in writing the manuscript.

## Conflict of Interest

The authors declare that the research was conducted in the absence of any commercial or financial relationships that could be construed as a potential conflict of interest.

## Footnotes

^1^In this work we consider the following dimensions: time is in [μ*s*], the resistors expressed in [*kΩ*], the currents are in [*A*], voltages are in [*mV*] and capacities are expressed in [*nF*].

^2^The circuit implementation of diffusive coupling is realized by linear resistors *R*_*ik*_ connecting the *ith* and *kth* MOCs through *x*_*i*_ and *x*_*k*_.
